# Epidemiologic and Clinical Profiles of Breast Diseases in Niger

**DOI:** 10.15436/2377-0902.15.015

**Published:** 2015-07-17

**Authors:** Harouna Zouladeny, Issimouha Dille, Nizar K Wehbi, Jungyoon Kim, Amr S. Soliman

**Affiliations:** 1 Department of Health Services Research & Administration, University of Nebraska Medical Center College of Public Health, Omaha, NE; 2 Niamey National Hospitals, Surgical Oncology Department, Niamey, Niger; 3 Department of Epidemiology, University of Nebraska Medical Center College of Public Health, Omaha, NE

**Keywords:** Breast cancer, Breast diseases, Epidemiology, Niger, Sub-Saharan Africa, Male breast cancer

## Abstract

This study aimed at characterizing epidemiologic and clinical profiles of breast diseases in Niger during the period of 2010–2013 at the National Hospital of Niamey. Medical records were abstracted for demographic, reproductive, clinical, and treatment information. A process map of patient navigation and barriers to seeking medical care was developed after interviewing 26 local health professionals who encounter and/or manage breast diseases. We identified 245 breast cancers and 122 other breast diseases. Mean age of breast cancer patients was 45.4 (±13.26 years) and that of breast diseases was 31(±12.5 years) with 1/3 of cancers under age 44. Infection-related diseases represented 24% of non-cancers. Male breast diseases represented 4.75% of diseases and 2.05% of cancers. Only 37.1% of cancers had histopathologic confirmation and 90% of cancer patients presented at advanced stages and mastectomy was performed for 66% of breast cancers. Patient and system barriers to care were common in diagnosing and treating breast diseases. Women in Niger have double burden of infectious breast diseases and emerging breast cancer. Younger age and late diagnosis are common features. Reducing barriers to access to care, down-staging of cancer, implementation of clinical guidelines for managing advanced cases are important needs for reducing breast cancer morbidity and mortality in Niger.

## Introduction

Sub-Saharan African countries are experiencing a double burden of infectious and chronic diseases^[1,2]^. Limited sanitation and prevalent infections coupled with aging and westernization contribute to the double burden of infectious and chronic diseases, such as cancer^[3,4,5–7]^. Women in developing countries suffer both breast diseases and breast cancer but with limited disease registration, the burden of these conditions and their management are difficult to characterize^[4]^. Niger represents one of the low-income African countries^[8]^ that have little information about breast diseases or cancer. Previous small-scale studies from Niger outlined some demographic and histopathologic characteristics of breast cancer^[9–11]^ but no recent studies have addressed breast diseases or breast cancer and their management. This study aimed at characterizing the epidemiologic and clinical management profiles of breast diseases and breast cancer at the National Hospital of Niamey, the largest general hospital in the capital of Niger. The study also aimed at outlining the characteristics of the local health system that might relate to the clinical and management profiles of these breast conditions.

## Methods

### Study Population and Data Sources

This study was conducted at the Niamey National Hospital (NNH) in Niamey, the capital city of Niger during the period of March to August 2013. The study included all patients with breast diseases and breast cancer diagnosed and/or treated at the NNH between January 2010 and August 2013. The NNH is level III national referral hospital where most of breast cancer patients are referred by physicians or hospitals or self-referred from different parts of the country.

The NNH cancer registry and logbooks of the departments of surgery and oncology for the period listed above were reviewed to identify medical record numbers of patients with any breast disease condition. Medical records were retrieved and data was abstracted on demographic and reproductive factors (age, sex, marital status, place of residence, and parity) disease characteristics and management (symptoms and signs, duration of illness, histopathologic features, and surgical and/or chemotherapeutic treatment).

### Data Management and Analysis

**Figure 1:**
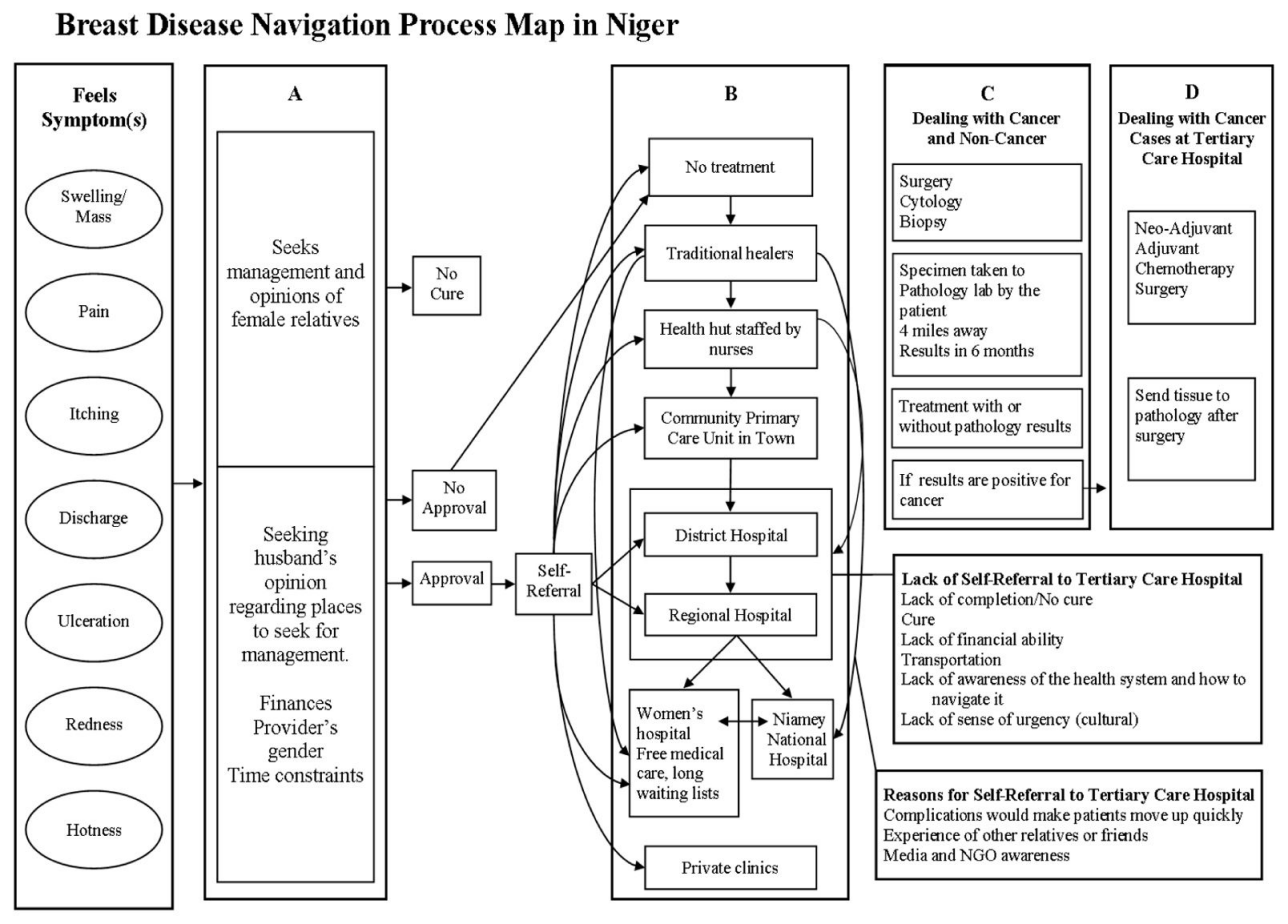
Depicts symptoms, navigation patterns, and different levels of provision of medical care for breast disease patients in Niger. As shown in the figure, patients seek opinions and approvals from influential family members (e.g. husbands, mothers, and grandmothers). Based on the opinions and decisions of these relatives, patients may seek medical care at traditional healers, primary care levels, or district hospitals. Based on the extent of symptoms, acute or chronic status of the lesions, availability of financial support, and transportation, patients may be referred or self-referred to treatment centers.

Frequency distribution of all the variables that were abstracted (age, sex, marital status, parity, place of residence, disease symptoms and signs, histopathologic features, surgical/chemotherapeutic management) was performed. Comparisons of categorical variables (the association between types of treatment and breast cancer, histopathology and menopause/chemotherapy and other treatment) were performed using the Mantel Haenszel chisquare tests and Fisher’s exact test. Symptoms and signs as well as management procedures were analyzed by cancer vs non-cancer diseases status. Breast cancer and non-breastcancer were categorized based on the confirmed histopathologic results. However, we analyzed breast cancer status based on confirmed histopathologic diagnosis of cancer and/or treatment with chemotherapy with or without surgery. A p-value <0.05 was considered statistically significant and SAS 9.3 (SAS, Cary, statistical package was used for the statistical analysis. From the results of the statistical analysis and the literature review, we realized that a large proportion of patients were seen at the study hospital at advanced disease stages. Therefore, we conducted interviews with 26 health professionals including treating physicians at the study hospital and referral primary care clinics in Niger. The interviews focused on the processes of patient navigation experiences in consultation, diagnosis, treatment, and follow-up of breast diseases. Based on the interviews, we developed a process map for navigation of breast disease patients in Niger from early symptoms and signs to diagnosis to treatment. This map could help in better understanding of late stage presentation and barriers and factors related to breast diseases management (Figure 1). Process mapping is one of the widely used analytical techniques in identifying relationships between activities, patients, data, and objects involved in the service delivery processes^[12]^. In health care management, this tool helps in understanding the entire patient journey or pathways with a range of individuals who represent the different roles of providers involved in health care delivery. Process mapping provides opportunities for improvement by visualizing how the whole patient navigation works and identifying points of inefficiency. By capturing duplication, variation, and unnecessary steps, the process map inspires the health care team to generate new ideas and help them to recognize where to start for making improvements that brings the biggest impact for patients and health professionals^[13]^.

The study was approved by the Institutional Review Boards of the University of Nebraska Medical Center and the NNH.

## Results

The study included 367 patients diagnosed with breast diseases, including breast cancer, at the NNH during the study period. These patients included 245 cancer patients, 122 non-cancer breast disease patients. The mean age was 45.4 (±13.26 years) for the breast cancer patients and 31.0 (±12.5 years) for the non-cancer breast disease patients (Table 1). Male breast diseases represented (4.75%) of non-cancer breast patients and 2.05% of breast cancers. Around 80% of the breast diseases patients lived in Niamey or relatively closer (<150 km), and only less than 7.9% of the patient came from a distant regions (>300km) p=0.05 (Table 1).

Histopathologic types of breast cancers were available for about half cases (183 cases) and they were infiltrating ductal carcinoma 54 (22.04%), infiltrating lobular carcinoma 14 (5.71%), metaplastic carcinoma 6 (2.45%) and other types of breast cancer accounted for 19 cases (9.39%) (Table 2). Type of information on non-breast cancer was in the following order: fibroadenomas 50 (54.35%), mastitis 15 (16.30%), breast abscess 7 (7.61%) and other breast diseases 20 (21.73%) (Table 3).

The high proportion of missing data for the number of children (191 cases), marital status (190 cases) and duration of symptoms (211 cases) limited our ability to draw a conclusion about the relationship between these variables and breast cancer. Breast cancer was slightly prevalent in the left side of the breast, 82 cases (24.83%) than the right side of the breast, 72 cases (21.36%).

In more than half the cases, tumor mass was reported for 131 cases (38.87%). Peaud’s Orange appearance was reported for 14 cases (4.15%) and the presence of axillary lymph nodes was reported in 24 cases (7.12%). Others signs and symptoms such as pain, ulceration, and erythema (redness) were recorded in an insignificant proportion of records.

Type of treatment was depicted in Table 4. Surgery was the most used method of treatment for 142 cases (42.14%) including 104 (30.86%) mastectomies. Chemotherapy alone was given to 79 patients (23.44%) and combination of chemotherapy and surgery treatment was provided for 84 patients (24.93%). Mastectomy and lumpectomy or lymph node removal was reported in medical records of 61 cases (18.10%), adjuvant chemotherapy for 129 cases (38.28%), and neo-adjuvant therapy for only 11 cases (3.26%) (Table 5).

The relationship between histopathologic characteristics, menopausal status, and chemotherapeutic and other treatments is presented in Table 5. No association was found between histopathologically-diagnosed breast cancer and chemotherapy treatment (p=0.5). Similarly, no association was found between menopausal status and histopathologic diagnosis of breast cancer, p=0.39. However, more histopathologically-diagnosed breast cancers were treated with chemotherapy and surgery, 57 cases (23.27%), p=0.0001 (Table 5).

## Conclusions

This study revealed a few interesting observations about breast diseases, including cancer, in Niger.

- A significant proportion of breast cancer cases are diagnosed among young patients under age 44.
- A limited proportion of breast cancer patients are diagnosed by histopathologic examination of biopsy specimens and the vast majority of suspected cancer cases are treated with chemotherapy without histopathologic confirmation.
- Surgery is the main method of breast disease treatment in this study hospital.
- Male breast cancer is not uncommon among breast disease patients.
- A high rate of infection-related breast diseases contributes to a significant proportion of breast diseases.
- Patient and system barriers to care are common features in the diagnosis and treatment of breast diseases in this population.

Regarding the young onset of breast cancer observation, this finding was similar to those of other studies from low-income countries. Nearly 30% of breast cancer patients in a study from Nigeria were under age 40^[14]^. Likewise, African immigrants to Sweden also had a mean age of approximately 44 years when diagnosed with breast cancer compared to their Swedish counter parts^[15]^. Before concluding that this population in Niger experiences a young-onset breast cancer pattern, calculating age-specific rates and accounting for the large population of young populations at the young age groups should be considered. Unfortunately, because of lack of systematic resources of cancer registration and limited information about the population denominator, young onset pattern could be a reflection of the large strata of the general population of women at young age in Niger.

In this study from Niger, about 90% of breast disease patients receive medical attention late in the course of disease. This is consistent with findings of a previous study from Niger that found more than 90% of patients during late stages of disease^[9]^. Large proportions of patients presenting at late stages were reported in other Sub-Saharan countries such as Senegal (60%), Cameroon, and Nigeria (58.3%)^[16–18]^. Although the proportions from these countries were high, the proportion found in our study was much higher. In a previous study from Niamey, breast cancer constituted 16.51 % of all cancers^[11]^. However, this proportion might be much higher if all the cases of breast cancers in the country are detected.

In addition to the morbidity and mortality implications of advanced stage at diagnosis, advanced-stage breast cancers are difficult to treat and costly in management even when treatment is available in low-income countries^[19]^. With the advanced stage at diagnosis of breast cancer in this study, it is possible that many other cases of neglected and undetected breast cancersdie in remote areas without diagnosis or treatment.

Histopathologic confirmation of the resected or biopsied lesions plays an essential role in confirming the diagnosis and planning the appropriate treatment of cancers. Previous research has shown that sub-Saharan African countries have 1/10 of the needed pathology services^[20]^. The lack of pathology services is due to a combination of factors varying from little monetary resources to inadequately trained personnel^[20]^. For example, in Sierra Leone, there are only 3 pathologists for 5 million people and in Uganda there are 18 pathologists for 28 million residents. Furthermore, these services, if available, are generally found only in tertiary-level hospitals^[21]^. Even when under-staffed pathology laboratories exist in low-income countries, they face additional logistical challenges of intensive paperwork, lack of space for storing specimens and paraffin blocks, lack of resources for maintenance of lab equipment, and lack of reagents^[22]^.

Patients in Niger are generally offered chemotherapy in two major hospitals despite the lack of histopathologic confirmation. The waiting time for receiving treatment could reach several weeks to months. Chemotherapy treatment is very expensive and out of reach for many patients in Niger ($60 to $140 pertreatment cycle). Furthermore, the poverty level in the country is so high that many women are unable to get access to breast cancer chemotherapy treatment, even when chemotherapy is paid for by charity and donations (Dille, personal communication, August 18, 2013).

No surgical oncologists are available in Niger except at NNH and Maternity Issaka Gazoby (MIG) hospital in Niamey. The majority of breast cancer patients in the country are treated by general surgeons before possible referral to NNH and MIG. Because of the limited access and lack of affordability of chemotherapy in addition to limited knowledge of general surgeons about comprehensive cancer management, surgical resection of lesions is the predominant method of treatment. Even at NNH and MIG, patient navigation is difficult, as patients could directly consult with the surgery department or chemotherapy instead of following a standard patient admission protocol: surgery followed by chemotherapy and radiotherapy. It is important to note that radiotherapy treatment is not available in Niger.

Our study identified a higher rate of male breast cancer than rates in non-African populations. A previous study from Niger reported a 5.7% breast cancer rate among males^[10]^. Higher rates of approximately 9% male breast cancer were reported from Nigeria^[23–24]^. Studies from North Africa also reported that male breast cancer accounts for nearly 1.4%–2.3% of all breast cancers^[25]^. Similarly, nearly 3% of breast cancers in Tanzania were found among males^[26]^. Studies suggest that African-Americans and native Africans may have higher susceptibility to breast cancer, possibly due to mutations in the BRCA genes^[27]^.

Another interesting finding of our study was the occurrence of high rate of infection-related breast diseases. It is possible that exposure to tuberculosis, HIV, and infectious agents such as *Staphylococcus aureus* can lead to breast diseases related to infection^[28,29]^. Without proper treatment, chronic breast lesions could develop and add to the burden of breast diseases in Niger. It is important to note that developing countries, such as Niger, are experiencing the double burden of infectious as well as non-communicable diseases^[1,2]^. This is clear from this study, where the study hospital is experiencing the double burden of infectious as well as non-infectious breast diseases.

The picture of healthcare management in Niger has shown that several bottlenecks prevent breast disease and breast cancer patients from getting access to proper care. Within the Niger healthcare system, there is no clear breast disease or breast cancer referral pattern due to quasi non-qualified health care providers, absence of cancer centers, and expensive costs of chemotherapy. One of the first reactions for women suffering from a breast disease is to seek the advice of a family member. This could be followed by consulting a traditional healer. After unsuccessful results, these women are referred or self-referred to local health huts, or primary healthcare clinics, or larger provincial or district hospitals. Because of the lack of medical expertise on breast diseases, most of the healthcare professionals provide insufficient or incomplete treatment for the disease.

This study had a number of strengths. This was one of the few studies from Niger recording the epidemiologic and clinical profiles of breast diseases over a 4-year period in the largest hospital in the capital city of Niger. However our analysis was limited by missing data for a number of variables due to the incompleteness of medical records and lack of a proper hospital cancer registry. Improving quality and completeness of medical records could enhance future research in this hospital and population of Niger.

Overall this study draws attention to the situation of breast diseases, including cancer, in Niger who mostly present at young age, advanced disease stages, and limited health system for diagnosis and treatment. In addition the study reported significant proportion of infection-related breast diseases. Hormone receptor determination of tumors was not available for the majority of cases and radiotherapy does not exist in the country of Niger. In a low income country like Niger, the implementation of a nurse navigator system similar to midwives in maternal care, may improve access and quality of breast disease management. Improvement and expansion of cancer care facilities in addition to increasing the number of qualified healthcare providers, increasing awareness of women regarding breast cancer, improving palliative care, and increased research related to reduction of barriers to care and downstaging are essential factors for breast cancer prevention and control in Niger and other similar low and middle-income countries. The process map should be utilized for planning future directions, setting priorities as well as strategies, and seeking financial and technical support by the government of Niger.

## Figures and Tables

**Table 1:** Characteristics of the study population

Characteristics	Non cancers		Cancers		p-value
	N = 89	%	N=236	%	
Age at Diagnosis(325,337)					
14–24	37	41.57	14	5.93	
25–34	20	22.47	37	15.68	0.0001
35–44	18	20.22	61	25.85	
45–54	10	11.24	64	27.12	
55+	4	4.49	60	25.42	
Mean Age(StD) years	31(12.5)		5.4(13.26)		
Sex(337,337)					
Male	9	9.78	7	2.86	0.05
Female	83	90.22	238	7.14	
Residence(177,337)					
Niamey	17	65.38	71	47.02	
<130km from Niamey	7	26.92	66	43.71	0.21
>300km from Niamey	2	7.69	14	9.27	
Reproductive(Parity)					
Number of Children(69,337)					
No children	6	40.0	17	31.48	
1–2 children	3	20.0	16	29.63	
3–5 children	2	13.33	16	29.63	
6+	4	26.67	5	9.26	
Marital Status(80,337)					
Divorced	2	8.0	6	10.91	
Widow	1	4.0	4	7.27	
Married	22	88.0	45	81.82	
Duration of Breast Diseases Symptoms(47,337)					
Three Months or less	3	23.08	8	23.53	
4 to 10 Months	4	30.77	10	29.41	
1 year+	6	46.15	16	47.06	
Side of Lesion(248,337)					
Bilateral	16	18.18	6	3.75	
Left	33	37.50	82	51.25	
Right	39	44.32	72	45.00	

**Table 2:** Histopathologic type of breast cancer lesions

Breast Cancer type	N=245	%
Anaplastic	1	0.41
Ductal carcinoma	3	1.22
Infiltrating Lobular carcinoma	14	5.71
Infiltrating ductal carcinoma	54	22.04
Infiltrating micropapillary carcinoma	3	1.22
Malignant lymphoplasmocytic lymphoma	1	0.41
Malignant phylloid tumor	4	1.63
Metaplastic carcinoma	6	2.45
Paject disease of breast	1	0.41
Unspecified breast cancer	154	62.86
Lobular carcinoma in situ	2	0.82
Mixed ILC-IDC	2	0.82

**Table 3:** Histologic type of non-cancers lesions

Non Breast Lesions	N=92*	%
Breast abscess	7	7.61
Breast sclerosis	1	1.09
Duct ectasia	2	2.17
Fibroadenomas	50	54.35
Fibrosis and simple cysts	4	4.35
Gynecomastia	5	5.43
Lipoma	5	5.43
Mastitis	15	16.3
Phylloid tumor	3	3.26

* 30 patients had no specific types of breast disease recorded in the medical records

**Table 4:** Type of Treatments by diagnosis

Type of Treatments	Cancer		Non Cancer		p-value
	245	%	92	%	
Chemo only	79	32.24	0	0	0.0001
Chemo and Surgery	84	34.29	3	3.26	
No treatment or unknown	24	9.80	21	22.83	
Surgery only	58	23.67	68	73.91	
**Surgery**					
Surgery	142	57.97	71	77.17	**0.001**
No known Surgery	103	42.04	21	22.83	
**Other Treatments**					
Lumpectomy or removal of lymph nodes only	8	6.40	35	67.31	**0.0001**
Mastectomy only	56	44.80	17	32.69	
Mastectomy and lumpectomy or removal of Lymph nodes only	61	48.80	0	0	
**Chemotherapy**					
Chemo	163	95.88	3	75	
No Chemo	7	4.12	1	25	
**Neo-Adjuvant**					
Neo-Adjuvant	11	7.69	0	0	
No Neo-Adjuvant	132	92.31	1	100	
**Mastectomy**					
Mastectomy	104	42.45	16	17.39	
No Mastectomy	141	57.55	76	82.61	

**Table 5:** Distribution of Treatment Types by Presence of Absence of Histopathologic Results

Menopausal status	Histopathology		No Histopathology		p-value
	N=91	%	N=145	%	
Yes	46	50.55	65	44.83	.04
No	45	49.45	80	55.17	
Chemotherapy					
Yes	64	96.52	99	96.12	.053
No	3	4.48	4	3.88	
Other Treatments					
Chemo Only	7	7.69	72	46.75	
Chemo and Surg	57	62.64	27	17.53	<.0001
No treatment or Unknown	7	7.69	17	11.84	
Surgery Only	20	21.98	38	24.68	

## Abbreviations

IBC: Inflammatory Breast Cancer
NNH: Niamey National Hospital
ER: Estrogen Receptor
PR: Progesterone Receptor
MBC: Male Breast Cancer
B: Bilateral
L: Left
R: Right
